# Survival Prediction Score: A Simple but Age-Dependent Method Predicting Prognosis in Patients Undergoing Palliative Radiotherapy

**DOI:** 10.1155/2014/912865

**Published:** 2014-03-19

**Authors:** Kent Angelo, Astrid Dalhaug, Adam Pawinski, Ellinor Haukland, Carsten Nieder

**Affiliations:** ^1^Department of Oncology and Palliative Medicine, Nordland Hospital, 8092 Bodø, Norway; ^2^Institute of Clinical Medicine, Faculty of Health Sciences, University of Tromsø, Tromsø, Norway

## Abstract

*Purpose*. Validation of a Canadian three-tiered prognostic model (survival prediction score, SPS) in Norwegian cancer patients referred for palliative radiotherapy (PRT), and evaluation of age-dependent performance of the model. *Patients and Methods*. We analyzed all 579 PRT courses administered at a dedicated PRT facility between 20.06.07 and 31.12.2009. SPS was assigned as originally described, That is, by taking into consideration three variables: primary cancer type, site of metastases, and performance status. *Results*. Patients with poor prognosis (non-breast cancer, metastases other than bone, and Karnofsky performance status (KPS) ≤ 60) had median survival of 13 weeks. Those with intermediate prognosis (two of these parameters) survived for a median of 29 weeks, and patients with good prognosis for a median of 114 weeks, *P* < 0.001. While this model performed well in patients who were 60 years or older, it was less satisfactory in younger patients (no significant difference between the good and intermediate prognosis groups). *Conclusion*. SPS should mainly be used to predict survival of elderly cancer patients. However, even in this group accuracy is limited because the good prognosis group contained patients with short survival, while the poor prognosis group contained long-term survivors. Thus, improved models should be developed.

## 1. Introduction

Gradual refinement of palliative oncological treatment approaches has contributed to better, prognosis-adapted cancer care. In part, disease trajectories extend over many years, even in patients without curative treatment option. In contrast, other patients with poorly responding tumors often face rapid disease progression and, as a direct consequence, limited survival. Clinicians are trying to tailor their treatment approaches by estimating patients' prognosis. While physicians' clinical experience and previous course of disease might provide hints [1, 2], a large number of more objective assessments tools have been developed [3–8], both for research purposes, patient stratification in clinical trials, and decision making. The choice between different tools might not always be easy. Ideally, prognostic scores are easy to administer, without need for expensive imaging or biomarker assessment, and valid across different institutions and countries [9]. The survival prediction score (SPS) developed by Chow et al. is among the tools that might be widely applicable, because it is based on three readily available parameters: primary cancer type, site of metastases, and performance status [10]. The present study reexamines its usefulness in patients treated with palliative radiotherapy (PRT), a widely used treatment modality. Given that other prognostic models often include age, we were interested in testing the SPS in separate patient groups with younger and older age, respectively.

## 2. Methods

We retrospectively reviewed the records of 412 consecutive patients who received one or more courses of PRT at a single hospital with dedicated PRT unit (Nordland Hospital, Bodø, Norway (an academic teaching hospital, which is the only provider of radiation oncology services in the county of Nordland)). The patients started their treatment in the time period from June 20, 2007 (date of opening of the dedicated PRT unit) to December 31, 2009 (this date was chosen in order to allow for sufficient followup of potential long-term survivors). A total of 579 courses were studied (299 patients received only one course of PRT, 78 patients received two courses, 24 patients received three courses, and 11 patients received 4–6 courses). Stereotactic radiotherapy was not included in the present series. All medical records, treatment details, and information on date of death were available in the hospital's electronic patient record (EPR) system. The survival status and date of death or last followup of the patients were obtained from the EPR. Patients who were lost to follow up were censored on the date of last documented contact (personal appointment, telephone conversation, and blood test). Patients who started a new course of PRT after their first one were censored on day 1 of the new course. This was done repeatedly if several PRT courses were administered to the same patient, because each course requires a new estimate of prognosis in order to avoid inappropriate under- or overtreatment. Median followup for all censored patients was 207 days. Survival time was measured from start of PRT. Actuarial survival curves were generated by Kaplan-Meier method and compared by log-rank test (analyses performed with IBM SPSS Statistics 20). We assigned SPS as described by Chow et al., that is, based on three variables (nonbreast cancer, metastases other than bone, and Karnofsky performance status (KPS) ≤ 60) [10]: poor prognosis group, when all three were present, intermediate prognosis group, when two were present, and good prognosis group, when 0-1 were present. We decided to dichotomize our patient group at an age cutoff of 60 years, because other prognostic models relied on this particular age limit [8, 11–16].

## 3. Results

**Figure 1:**
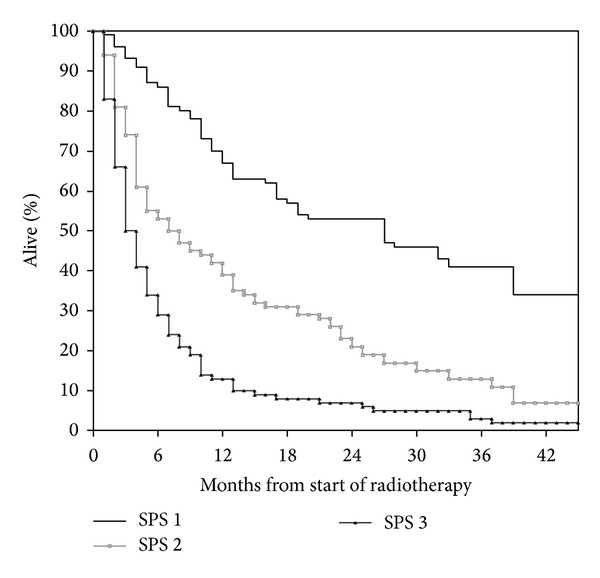
Actuarial overall survival after palliative radiotherapy (Kaplan-Meier estimate): median 114 versus 29 versus 13 weeks; *P* < 0.001. Number of patients in each group: 177, 145, and 254.

**Figure 2:**
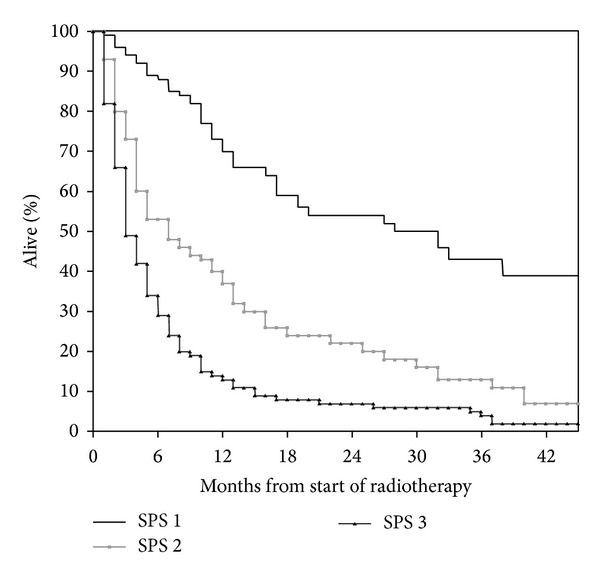
Actuarial overall survival after palliative radiotherapy in patients aged 60 years or older (Kaplan-Meier estimate): median 68 versus 18 versus 10 weeks; *P* < 0.001. Number of patients in each group: 145, 117, and 218.

**Figure 3:**
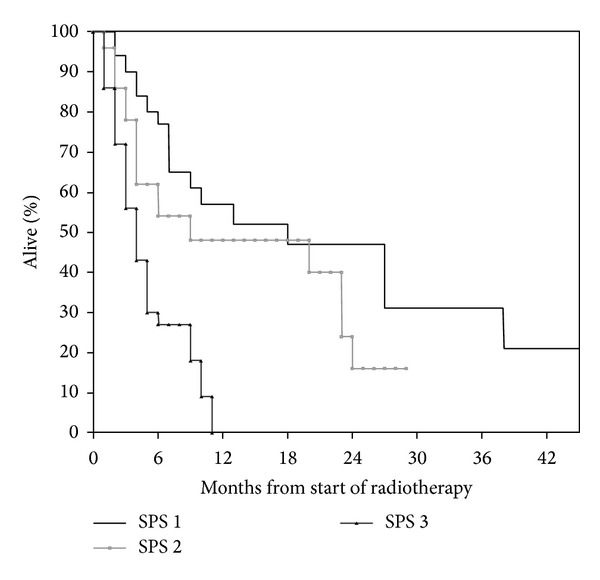
Actuarial overall survival after palliative radiotherapy in patients younger than 60 years (Kaplan-Meier estimate): median 39 versus 83 versus 16 weeks, no significant difference between groups 1 and 2. Number of patients in each group: 32, 28, and 36.

Median age at the time of PRT was 70 years (range 31–97 years). Prostate (25%) and nonsmall cell lung cancer (NSCLC, 18%) were the most common primary tumors. Median time interval from first cancer diagnosis to PRT was 27 months (range 1–386 months). Additional baseline information is shown in Table 1. Bone metastases were the prevailing target for PRT (54%). The most common PRT regime consisted of 10 fractions of 3 Gy (36%). Other common regimes included 8 Gy single fraction (uncomplicated bone metastases), 2 fractions of 8.5 Gy (thoracic PRT for lung cancer), and 5 fractions of 4 Gy (various sites and indications). Twenty-five PRT courses (4%) remained incomplete, typically because of earlier than expected clinical deterioration. Median survival from PRT was 27.6 weeks (6.3 months). Essential information required to assign SPS was lacking in three patients. For all remaining 576 patients, SPS significantly predicted survival (Figure 1; *P* < 0.001). However, long-term survival was observed even in the unfavorable subgroup with three adverse features (nonbreast cancer, metastases other than bone, and poor performance status). Nineteen percent of patients with these poor SPS features died within 30 days. As shown in Figure 2, SPS significantly predicted survival of patients with age 60 years or older; *P* < 0.001. Long-term survival was observed in all three subgroups. As shown in Figure 3, SPS performed less satisfactory in younger patients. On the one hand, the unfavorable subgroup did not contain long-term survivors. On the other hand, the two subgroups with better prognosis had almost undistinguishable survival. The patient numbers were more equally distributed between the three prognostic subgroups in younger patients (maximum group size 37.5%). As in their younger counterparts, the largest subgroup of older patients had poor prognostic features (45%). Overall, age did not significantly influence median survival (28.6 weeks in younger and 27.4 weeks in older patients; *P* = 0.65).

## 4. Discussion

Development of the Chow et al. model started in 395 patients referred to their PRT program [5]. Later, they refined their original six-parameter model by reducing the number of variables to three, arriving at the SPS [10, 17]. The latter study contained three patient cohorts with median age of 66, 68, and 69 years, respectively. Two cohorts of patients treated in 2000 and 2002 were included for validation purposes, while the training set consisted of patients treated in 1999. Compared to our data (2007–2009), median survival was worse in all three cohorts and all three prognostic groups. While we reported a median survival of 114 weeks in the most favorable group, Chow et al. reported 55–64 weeks. In the intermediate group, we found a median survival of 29 weeks, whereas Chow et al. found median survival of 19–28 weeks. In the unfavorable group, the corresponding results were 13 versus 9-10 weeks. Possible explanations might include different PRT timing (lead time bias), improved diagnostic imaging (stage migration), and better systemic therapy resulting in truly improved survival. In their study, 14% of patients were assigned to the good, 40% to the intermediate, and 46% to the poor prognosis groups. These results compare to 31% with good, 25% with intermediate, and 44% with poor prognosis in the present study. Our data confirm the validity of the SPS in more recently treated patients from a different geographical region. Chow et al. found that the amount of explained variability was low, that is, <30%, which means that it is unrealistic to predict survival with a high degree of accuracy when only three parameters are taken into consideration [10]. This can also be seen in Figure 1, showing that the good prognosis group contained a proportion of patients with short survival, while the poor prognosis group contained some long-term survivors. For the first time, we reported that SPS gives age-dependent results, making this score less applicable to patients who are younger than 60 years (Figure 3).

Disadvantages of our study include its retrospective design and the facts that patient numbers were limited, especially regarding subgroups, and that most patients were elderly (median age is 70 years). The majority of PRT courses consisted of hypofractionated regimens, mostly 1–15 fractions, with dose/fractionation parameters reflecting a patient's expected prognosis (clinical estimate). We did not use any particular prognostic models or scores when assigning treatment regime. One of the clinical advantages of validated prognostic scores could be reduced overtreatment in patients with very short survival [18]. Recently, Guadagnolo et al. have reported on the use of radiotherapy in the last 30 days of life in the United States [19]. They used a SEER-Medicare linked database to obtain a large study cohort of 202,299 patients who died as a result of lung, breast, prostate, and colorectal and pancreas cancers (top five cancer causes of death) between January 1, 2000, and December 31, 2007. The rate of radiotherapy in the last 30 days of life, by many regarded as inappropriate overtreatment, was 7.6%. No attempt was made to develop predictive models. Our results indicate that SPS is not suitable for prediction of very short survival, for example, death within one month from PRT. Nineteen percent of patients in the poor SPS group died within 30 days. In other words, one would withhold potentially useful treatment in a large number of patients when completely forgoing PRT in the subgroup with three adverse SPS features. It is therefore important to develop better decision tools that facilitate tailored palliative approaches. Recent research suggests that more advanced tools might perform better than unspecific approaches such as SPS. For example, Rades et al. have developed scores specific to metastatic spinal cord compression [20, 21], Sperduto et al. to brain metastases [22], and our own group to prediction of very short survival in patients with brain metastases [23]. Future research will probably focus on rather narrowly defined patient groups, taking into account not only primary tumor type and site of metastases but also histological or molecular subgroups. Such information is increasingly being used to decide what type of systemic treatment should be prescribed [24].

## 5. Conclusion

In principle, SPS is a straightforward and easy-to-use prognostic score. However, it is important to realize its inherent limitations, especially with regard to younger patients. The range of potential survival outcomes in each prognostic group is large, confining its contribution to decision making in individual patients outside of study protocols.

## Figures and Tables

**Table 1:** Patient characteristics.

Characteristic	Number	%
Entire cohort	579	
Age (years)		
<60	96	16.6
≥60	483	83.4
Gender		
Male	356	61.5
Female	223	38.5
Karnofsky performance status^1^		
90–100	75	13.0
70–80	160	27.6
≤60	343	59.2
Primary tumor site		
Prostate	145	25.0
Breast	67	11.6
Lung (small cell)	31	5.4
Lung (nonsmall cell)	105	18.1
Colorectal	37	6.4
Pancreas	9	1.6
Bladder	32	5.5
Other	153	26.4
Number of RT fractions		
1–4	114	19.7
5–9	140	24.2
10	210	36.3
11–15	90	15.5
>15	25	4.3
Dose per fraction (Gy)		
<3	60	10.4
3	262	45.3
3.1–3.9	16	2.8
4	125	21.6
4.1–5	26	4.5
>5	90	15.5
Selected target types		
Bone metastases	314	54.2
Brain metastases	68	11.7
Lymph node metastases	34	5.9
Brain metastases^1^		
No	483	83.4
Yes	92	15.9
Liver metastases^1^		
No	459	79.3
Yes	116	20.0
Lung metastases^1^		
No	451	77.9
Yes	124	21.4
Adrenal gland metastases^1^		
No	518	89.5
Yes	57	9.8
Bone metastases^1^		
No	194	33.5
Yes	381	65.8
Systemic cancer treatment^1^		
No	256	44.2
Within 4 weeks before RT	118	20.4
Within 3 months before RT	69	11.9
Earlier	79	13.6

RT: Radiotherapy.

^1^Missing information in some cases.
